# Prevention of Pregnancy Complications in Iran Following Implementing a National Educational Program 

**Published:** 2014-09

**Authors:** Maryam Moghani Lankarani, Nasrin Changizi, Mohammadreza Rasouli, Mohammad Amir AmirKhani, Shervin Assari

**Affiliations:** 1 Medicine and Health Promotion Institute, Tehran, Iran; 2 Ministry of Health and Medical Education, Tehran, Iran; 3 Department of Health Behavior and Health Education School of Public Health University of Michigan, MI, US

**Keywords:** National Program, Pregnancy Complications, Iran

## Abstract

**Objective:** To determine the impact of a national intervention program on some pregnancy complications in Iran.

**Materials and methods:** This multicenter study was conducted in governmental sector in 14 provinces in Iran between 2003 and 2005. Intervention included education of all maternal health care providers including gynecologists, general physicians, and midwifes in the governmental sector. Time interval between the pre- (of 3,978 and 3,958 pregnancies) and post- (3,958 pregnancies) measurements were 18 months. Self reported data on pregnancy complications were registered. Interviews were conducted by trained personnel. Participants were interviewed when admitted for delivery or at the time attending for vaccination of their 2 month infants.

**Results:** The following pregnancy complications were reduced significantly as compared to before intervention: 1) bleeding or spotting, 2) urinary tract complications, 3) blurred vision and severe headache, 4) premature labor pain, 5) anemia, 6) severe vomiting, 7) inappropriate weight gain, 8) endometritis, 9) urinary incontinence, 10) breast abscess or mastitis, 11) wound infection, and 12) bleeding was significantly reduced after intervention, compared to before intervention. Premature rupture of membrane showed a significant increase. These complications did not show a significant change: 1) hypertension, 2) fever and chills, 3) convulsion, shock, and loss of consciousness, and 4) obstetric fistula.

**Conclusion:** National programs may be proved to be largely effective by decreasing some of the pregnancy complications in developing countries.

## Introduction

Maternity care is to offer services that improve the health of pregnant women and their infants (1). The main goals of maternity care are to prevent, detect and treat complicated pregnancies. It is expected that women who are prone to complicated pregnancy can be identified to avert problems (2). High quality maternity care promotes favorable pregnancy outcomes (3).

Maternity care quality is a determinant of maternal mortality. Maternal education as a part of this care capacitates women to recognize and act on symptoms leading to potentially serious conditions (1). Maternity care is one of the important determinants of the rate of safe deliveries (4).

Improvements in maternal care are one of the Millennium Development Goals, developed by the World Health Organization (WHO) (5). Policy makers try to improve national maternal care indices by applying standard protocols and guidelines (6). 

In Iran, according to the Demographic and Health Survey (DHS), 2000, coverage of prenatal and postpartum care was 80% and 31%, respectively (7). Until 2000, maternal care services in Iran had major obstacles including absence of a standard protocol and not involving physicians in the maternal care services. Thus, the Maternal Health Center -the responsible center for policy making in maternal care- was established to set strategies and goals for the national maternal health system. The center designed and implemented a national outpatient maternal care development program in the governmental sector. 

In the current study we investigated the impact of this program on pregnancy outcomes within the first 18 months of its implementation.

## Materials and methods

This multicenter study was conducted in governmental health sector of 14 cities in different provinces in Iran from 2003 to 2005. Iranian Ministry of Health and Medical Education implemented the intervention, and also conducted the evaluation.

***Participants***

Before initiation of the program (2003), and also 18 months after it (2005), a total of 3978 and 3958 participants were enrolled, respectively. Participants were women who had been admitted in hospital or delivery facilitating units for delivery or attended for vaccination of their 2 month infants. Consecutive sampling was done.

***Ethical aspects***

Anonymous self-report questionnaire addressing confidentiality was used. The study was approved by the institutional review board at Ministry of Health and Medical Education, Tehran, Iran. All participants signed informed consent.

**Data collection**

The following 17 pregnancy complications measured in this study; 1) hypertension, 2) premature rupture of membranes, 3) bleeding or spotting, 3) urinary tract complications, 4) blurred vision and severe headache, 5) premature labor pain, 6) anemia, 7) severe vomiting, 8) fever and chills, 9) convulsion, shock and loss of consciousness, 10) inappropriate weight gain, 11) endometritis, 12) qnemia, 13) obstetric fistula, 14) urinary incontinence, 15) breast abscess or mastitis, 16) wound infection, and 17) bleeding.

Data collection was done by the personnel of the medical universities in each province. 

***ANC revision program***

The national program started at 2003 in all country. The program was implemented by maternal health center of minister of Health and medical education of the country. This program included the following goals: 1) reducing maternity visits (targeting from 14 visits to 8 visits), 2) involvement of physicians in maternity services, 3) minimizing un-necessary laboratory tests, 4) definition of a standard protocol for high risk and complicated pregnancy, 5) employing multidisciplinary approach by involving multiple administrative parts of the ministry, 6) definition of levels 1 and 2 of maternity care and a protocol for refereeing complicated pregnancy and 7) performing pilot evaluation before establishment of the designed program.

**Educational component**

The educational program used training for trainer (TOT) model. In the first step, a team consisted of members and experts of the scientific committee of the Maternal Health Center trained a team from each medical university. This trained team of the province composed of 1-2 gynecologists who were faculty members of that university plus 3 officers of Maternal Health Center of the province. In the second step, the trainees of each province acted as the trainers of all maternity health providers of their provinces (n=14). Maternity health care providers in the city included all gynecologists, general physicians, and midwifes.

***Quality control of the intervention***

In the first level, medical university authorities of each province supervised on the data collection and data quality control. In the second level, during the study period, external evaluation was performed by the Maternal Health Center, Ministry of Health and Medical Education. 

The program was monitored from 1 to 5 times in different cities. Overall quality of the educational process, structure and infrastructure of the running programs were evaluated in the provincial level. For the program monitoring, 10 quality indicators were employed. We did not report details of monitoring here, as the aim of this study was to present the impact of intervention. 

***Statistical analysis***

Statistical analysis was performed by STATA software (STATA Corp, USA) version 8.0. 95% of confidence intervals (95% CI) were calculated based on robustness method. Chi square test was used for comparison of the maternity care indicators before and after the program. P value < 0.05 considered statistically significant.

## Results

The following pregnancy complications significantly decreased following the intervention: 1) bleeding or spotting, 2) urinary tract complications, 3) blurred vision and severe headache, 4) premature labor pain, 5) anemia, 6) severe vomiting, 7) inappropriate weight gain, 8) endometritis, 9) urinary incontinence, 10) breast abscess or mastitis, 11) wound infection, and 12) bleeding. Premature rupture of membrane showed a significant increase during the intervention. These complications did not show a significant change: 1) hypertension, 2) fever and chills, 3) convulsion, shock, and loss of consciousness, and 4) obstetric fistula Premature rupture of membrane showed a significant increase (Table 1).

## Discussion

Our results showed the impact of a maternal care intervention program on several pregnancy complications in Iran. The changes included a decrease in bleeding or spotting, urinary tract complications, blurred vision and severe headache, premature labor pain, anemia, severe vomiting, inappropriate weight gain, endometritis, urinary incontinence, breast abscess or mastitis, wound infection, and also bleeding. These complications did not show a significant change: 1) hypertension, 2) fever and chills, 3) convulsion, shock, and loss of consciousness, and 4) obstetric fistula. Premature rupture of membrane showed a significant increase following the intervention.

American Academy of Pediatrics and American College of Obstetricians and Gynecologists have recommend that a woman with an uncomplicated pregnancy be examined every 4 weeks for the first 28 weeks of pregnancy, every 2 to 3 weeks until 36 weeks gestation, and weekly thereafter (8). However there is a worldwide tendency to reduce numbers of prenatal visits.

**Table 1:** Comparison of pregnancy complications before and after intervention

	**Before** **Mean (range)**	**After** **Mean (range)**	
Hypertension	6.5 (5.7-7.5)	6.2 (5.7-6.7)
Premature rupture of membranes*	2.4 (1.9-3)	4.8 (4.4-5.2)
Bleeding or spotting*	9.1 (8.1-10.2)	5.8 (5.5-6.1)
Urinary tract complications*	19.5 (18.1-21)	16.3 (15.5-17.1)
Blurred vision and severe headache*	7.2 (6.4-8.3)	3.3 (2.9-3.7)
Premature labor pain*	5 (4.3-5.9)	2 (1.7-2.3)
Anemia*	12.5 (11.4-13.8)	9.8 (9.2-10.4)
Severe vomiting*	6.1 (5.2-7)	3.8 (3.3-4.3)
Fever and chills	1.7 (1.3-2.3)	1.6 (1.3-1.9)
Convulsion, shock, and loss of consciousness	0.5 (0.2-1.4)	0.5 (0.3-0.7)
Inappropriate weight gain*	12.2 (11-13.4)	5.6 (5.1-6.1)
Endometritis*	18.3 (13.6-23.8)	2.4 (2.1-2.7)
Obstetric fistula	0.9 (0.1-3.1)	0.07 (0.04-0.1)
Urinary incontinence*	3 (1.2-6.1)	0.2 (0.1-0.3)
Breast abscess or mastitis*	9.8 (6.3-14.4)	1.6 (1.3-1.9)
Wound infection*	15.5 (11.1-20.7)	2.2 (0.9-3.5)
Bleeding*	17.6 (12.9-23.1)	2.2 (1.9-2.5)

* There were significant differences (p< 0.05) between the first and second assessments

Several studies have shown the safety of reduction of prenatal visits for uncomplicated pregnancy (9-13). Mc Duffie and associates suggested that good perinatal outcome is achievable with reduction in the numbers of maternity visits (11). In another study by Villar et al. in four countries including Argentina, Cuba, Saudi Arabia, and Thailand showed that a new maternity program with fewer number of visits (the median number of visits of 5) have not adverse affect on maternal and perinatal outcomes. Empirical evidence reveals that 4 maternity visits are sufficient for uncomplicated pregnancies and more are necessary only in cases of complications (9). Thus, the World Health Organization currently recommends a minimum of four maternity visits in the course of uncomplicated pregnancies (14).

Coverage of prenatal care is low in developing countries (15, 16). In China, 36% of pregnant women have fewer than 5 prenatal visits, and about 9% have no prenatal visits (15). The 1992–1999 Indian National Family Health Survey revealed that only 64-65% of Indian mothers participate in a maternity program (2). 

This study was partly based on self reported data and this may influence the accuracy of data. This intervention was only done in governmental sector, and is not representative to the all country. 

In conclusion, this report revealed promising impact of national program on pregnancy outcomes in the governmental health sector in Iran.
